# Achondroplasia Natural History Study (CLARITY): a multicenter retrospective cohort study of achondroplasia in the United States

**DOI:** 10.1038/s41436-021-01165-2

**Published:** 2021-05-18

**Authors:** Julie E. Hoover-Fong, Adekemi Y. Alade, S. Shahrukh Hashmi, Jacqueline T. Hecht, Janet M. Legare, Mary Ellen Little, Chengxin Liu, John McGready, Peggy Modaff, Richard M. Pauli, David F. Rodriguez-Buritica, Kerry J. Schulze, Maria Elena Serna, Cory J. Smid, Michael B. Bober

**Affiliations:** 1 Greenberg Center for Skeletal Dysplasias, Department of Genetic Medicine, Johns Hopkins University, Baltimore, MD USA; 2 McGovern Medical School, University of Texas Health, Houston, TX USA; 3 School of Dentistry, University of Texas Health, Houston, TX USA; 4 University of Wisconsin School of Medicine and Public Health, Madison, WI USA; 5 Nemours/A.I. duPont Hospital for Children, Wilmington, DE USA; 6 Bloomberg School of Public Health, Johns Hopkins University, Baltimore, MD USA; 7 Present Address: AYA: Bloomberg School of Public Health, Johns Hopkins University, Baltimore, MD USA; 8 Present Address: Children’s Wisconsin & Medical College of Wisconsin, Milwaukee, WI USA

## Abstract

**Purpose:**

Achondroplasia is the most common short stature skeletal dysplasia (1:20,000–30,000), but the risk of adverse health outcomes from cardiovascular diseases, pain, poor function, excess weight, and sleep apnea is unclear. A multicenter retrospective natural history study was conducted to understand medical and surgical practices in achondroplasia.

**Methods:**

Data from patients with achondroplasia evaluated by clinical geneticists at Johns Hopkins University, A.I. duPont Hospital for Children, McGovern Medical School UTHealth, and University of Wisconsin were populated into a REDCap database. All available retrospective medical records of anthropometry (length/height, weight, occipitofrontal circumference), surgery, polysomnography (PSG), and imaging (e.g., X-ray, magnetic resonance imaging) were included.

**Results:**

Data from 1,374 patients (48.8% female; mean age 15.4 ± 13.9 years) constitute the primary achondroplasia cohort (PAC) with 496 subjects remaining clinically active and eligible for prospective studies. Within the PAC, 76.0% had a de novo *FGFR3* pathologic variant and 1,094 (79.6%) had one or more achondroplasia-related surgeries. There are ≥37,000 anthropometry values, 1,631 PSGs and 10,727 imaging studies.

**Conclusion:**

This is the largest multicenter achondroplasia natural history study, providing a vast array of medical information for use in caring for these patients. This well-phenotyped cohort is a reference population against which future medical and surgical interventions can be compared.

## INTRODUCTION

Achondroplasia is the most common short stature skeletal dysplasia, with an estimated prevalence of 1 per 20,000–30,000 births.^1–3^ Clinical characteristics include short stature with relatively shortened limbs compared to trunk, rhizomesomelia with trident hand conformation, and macrocephaly with frontal bossing. Achondroplasia is caused by a gain-of-function pathologic variant in the fibroblast growth factor receptor 3 (*FGFR3*) gene, with one amino acid substitution (glycine-to-arginine at position 380) responsible for 98% of all cases.^4,5^ Approximately 80% represent a de novo variant in *FGFR3*^6^ on the paternal allele.^7^

There are distinct and recognizable—yet highly variable—skeletal manifestations in achondroplasia, including cervicomedullary compression, a generalized spondylometaphyseal dysplasia, and midface retrusion. For some, these skeletal abnormalities cause serious medical complications and necessitate invasive surgical treatment. For example, the foramen magnum is smaller than in average stature individuals in both transverse and sagittal dimensions (AP) and misshapen due to premature closure of cranial synchondroses.^8,9^ Surgical decompression of the foramen magnum and possibly the first (and sometimes second) cervical vertebrae is indicated when long-track signs on neurologic exam, central apnea identified by polysomnography (PSG), and/or signal changes in the cervical spinal cord by magnetic resonance imaging (MRI) are present.^10^ In the thoracic and lumbar spine, there is diffuse foraminal stenosis and decreased AP diameter causing neurogenic pain in nearly all individuals with achondroplasia.^11^ Surgical decompression is indicated when pain cannot be controlled, ambulation is severely compromised, or bowel/bladder dysfunction occurs. Metaphyseal irregularities and joint laxity contribute to a common genu varum deformity, resulting in leg pain and compromised physical function and may necessitate realignment osteotomies.^12^ Differential growth in regions of the face and cranium (e.g., frontal bossing concurrent with midface retrusion and mandibular prognathanism) contributes to obstructive sleep apnea (OSA).^13,14^ Surgical tonsillectomy, adenoidectomy, and/or continuous positive airway pressure (CPAP) is needed when the obstruction causes chronic hypoxia or hypercarbia during sleep.^15^ Despite recognition of these complications in achondroplasia, our understanding of their natural history is often limited by published experiences of a single case, small case series, and uncontrolled observational studies. Although valuable, data from a small population should not be generalized to all people with achondroplasia over the lifespan. Furthermore, there are no longitudinal studies of achondroplasia to assess the long-term efficacy of common surgical procedures.

A 42-year morbidity and mortality study showed higher than expected cardiovascular disease-related deaths among people with achondroplasia compared to age- and gender-matched average stature individuals,^16^ perhaps related to excessive central adiposity in the former.^17^ Excess weight exacerbates common achondroplasia-related medical problems including OSA, genu varum, spinal stenosis, and lumbar lordosis.^18^ Consistent with cardiovascular outcomes, elevated blood pressure is more common among individuals with achondroplasia compared to average stature adults in a recent study.^19^ Compromised mobility, chronic pain, and low physical activity also have been described.^20^ We need a comprehensive understanding of the natural history of achondroplasia, the most common dwarfing condition, to improve quality of life.

We established a well-phenotyped cohort of children and adults, the primary achondroplasia cohort (PAC), from four skeletal dysplasia centers (Johns Hopkins University, A.I. duPont Hospital for Children (AIDHC), McGovern Medical School UTHealth, and University of Wisconsin) to define the health risks and medical/surgical outcomes of patients with achondroplasia. We present natural history observations through a summary of surgical burden, growth curves comparing the PAC with age-matched average stature peers, the prevalence of sleep disordered breathing, and general health characteristics over time. This cohort is the source for more detailed cross-sectional analysis across these domains and longitudinal studies to understand the chronology and interactions of health complications in achondroplasia. The natural history of this cohort may be utilized as a baseline comparison for future studies of novel pharmacologic, medical, and surgical interventions.

## MATERIALS AND METHODS

### Participating sites and study population

Subjects were evaluated at least once between 1957 and 2017 by a clinical geneticist at one of four academic skeletal dysplasia centers in the United States: Johns Hopkins University (Baltimore, MD; coordinating site), AIDHC (Wilmington, DE), McGovern Medical School at the University of Texas Health (Houston, TX), and University of Wisconsin (Madison, WI). A diagnosis of achondroplasia was made by molecular or clinical means. There were no medical complications or conditions that otherwise excluded a patient. The PAC includes clinically active patients and those lost to follow-up but whose clinical records are available.

### Data collection and management

All available retrospective data were extracted from archives, current hard-copy charts, and electronic medical records to populate a REDCap database.^21^ This REDCap database is hosted on servers administered by the Data Informatics Services Core (DISC) of the Johns Hopkins Biostatistics Center. Uniform data collection forms ensured similar data collection at all study sites. Data entry errors or omissions were resolved by the primary study site until the database was locked in January 2019 after which changes were made by the coordinating site. The database was closed in May 2019 for this analysis. Data were monitored and cleaned throughout the entry process for potential inclusion of a subject at more than one site and for duplicate data.

The age of a subject reflects his/her age at the last known clinical encounter. All anthropometry was collected by multiple providers in the clinic setting. Length was obtained in a supine position until at least 2 years of age. Weight was obtained with a standard clinical scale and head circumference was measured by a physician or nurse in the dysplasia center with a tape measure. Algorithms also were applied to the data cleaning process of all anthropometry values (Supplemental Methods).

Medical records were examined for surgical procedures, date performed, indications, and outcome. There were several surgical procedures included under each of the five main categories: ear–nose–throat (ENT), brain, foramen magnum, spine, and extremities. Primary tonsillectomy/adenoidectomy and all revisions were included in the ENT category. Surgical placement of pressure equalizing (PE) tubes was included in the analysis only if a date of the procedure was available. In the brain surgery category, all occurrences of ventriculoperitoneal shunt placement and revision as well as ventriculostomy are included. The third surgical category involves enlargement of the foramen magnum with/without laminectomy of the first and sometimes second cervical vertebrae. These procedures are considered together in the foramen magnum category but separate from laminectomies and vertebral fusions of cervical vertebra 3 (C3) through the sacrum in the fourth category of spine surgery. The extremity surgery category included procedures to correct genu varum and lower extremity bowing.

All available PSG reports were included and standard PSG data fields were populated (Supplemental Methods). The final diagnostic determination of individuals with at least one sleep study was included in this analysis.

The final study domain was construction of a radiographic catalogue of all imaging (e.g., X-rays, computed tomography [CT] scans, MRIs). For each event, the study type, date performed, location of images, and availability of original images were recorded (since imaging reports are insufficient for further analysis).

### Analysis

Summary statistics and demographics of the PAC and active subcohort (i.e., sex, age at last encounter, study site, inheritance, adoption status, gestational age, prior limb lengthening, growth hormone deficiency/treatment and/or trial participation, and means and timing of diagnosis) by vital status and birth decade were generated. The total surgical procedures is presented as a proportion of the PAC population. For continuous measures, means and standard deviations, medians and interquartile ranges (IQR) and/or ranges are presented, and for binary and categorical measures, proportions are presented. The absolute number and proportion with at least one PSG, the proportion with moderate or severe OSA and the average age at the study by birth decade is reported.

Details of the anthropometry analysis are outlined in the Supplement Analysis. Subjects born before 37 weeks gestation (i.e., preterm) were omitted from the corresponding growth curves up to 2 years of age. Subjects of unknown gestation but with birth parameters within ±2 SD of the mean on published sex-specific achondroplasia growth curves were presumed to be term and their anthropometric values were included in the growth curves. Anthropometry from subjects who underwent limb lengthening, treated with growth hormone (with or without documented deficiency) and participants in a clinical trial of an investigational pharmaceutical to modulate linear growth were excluded from analysis.

Similar to prior publications,^22–24^ sex-specific percentile curves were generated in a two-step process for children with anthropometry data up to 18 years of age for stature and weight and from birth through 5 years for head circumference. Empirical 5th, 50th, and 95th percentiles were computed using values within a window of ±0.5 months for 0 to 12 months of age, ±1 month for >12 to 36 months of age, ±3 months for >36 to 120 months of age, and ±6 months for >10 to 18 years of age. All data were used to create isopleths for stature for age and head circumference for age for the 5th, 50th, and 95th percentiles. Because weight for age data skewness increased with increasing age, separate penalized smoothing splines were used to estimate the weight for age percentiles for birth to 3 years and 3 to 18 years. The height velocity at a given age was computed by taking the difference in two time-ordered length/height values (between 6 and 18 months apart), dividing this by the difference in ages of measurements, and plotting the quotient at the midpoint of the age difference. For all subjects, sex-specific mean height velocity values by age were computed. Sex-specific birth means, SD estimates, and 95% confidence intervals (CIs) were computed for length, weight, and OFC. The resulting CIs were compared to the corresponding average stature mean values from the Centers for Disease Control and Prevention/World Health Organization (CDC/WHO)^22,23^ to ascertain whether these differences were statistically significant.^25–27^

## RESULTS

This natural history registry includes retrospective data from 1,374 subjects with achondroplasia evaluated from 1957 to 2017. The PAC includes 1,354 living and 20 deceased individuals as of the last known encounter up to 1 January 2019 (Supplemental Table 1). The cause of death is unknown in 6 of the 20 deceased individuals. Of the 1,374 subjects in the PAC, 496 subjects continue to receive medical care from one of the four participating skeletal dysplasia clinical sites. General characteristics of the PAC and active subjects are presented in Table 1. Sex is distributed evenly in the PAC and active subcohort. The mean ± SD age (based on the last known clinical encounter) of the PAC is 15.4 ± 13.9 years (range 0–79.7 years) while the active subcohort is younger, with a mean age of 12.9 ± 12.2 (range 0–71.7 years). Achondroplasia was inherited in 13.9% of the PAC, de novo in 76.0%, and inheritance unknown in 10.1%. In the active subcohort, there are only 31 (6.3%) subjects in whom the inheritance pattern was unknown, indicating a de novo rate of 79%. In the PAC, 12.7% were born prematurely (<37 weeks gestation). A small proportion of the PAC had undertaken surgical or medical treatments to alter stature including 17 (1.2%) with limb-lengthening surgery, 4 (0.3%) with documented growth hormone deficiency by stimulation testing with 2 (0.1%) proceeding with treatment, and 12 (0.8%) individuals participating in recent clinical trials involving growth modulating research pharmaceuticals.

**Table 1:** Population characteristics of primary achondroplasia cohort (PAC) and active subcohort (active).

Characteristic		PAC		Active
	Total	Living	Deceased	Total
Participants, *n*	1,374	1,354	20	496
Sex, *n* (%)				
Male	704 (51.2)	696 (51.4)	8 (40.0)	256 (51.6)
Female	670 (48.8)	658 (48.6)	12 (60.0)	240 (48.4)
Age at last encounter, years				
Mean ± SD	15.4 ± 13.9	15.0 ± 13.3	39.7 ± 25.4	12.9 ± 12.2
Range	0.0–79.7	0.0–71.7	0.3–79.7	0.0–71.7
Median (IQR)	11.9 (5.6, 19.7)	11.8 (5.5, 19.4)	41.5 (18.1, 61.8)	9.1 (4.3, 16.8)
Clinical site, *n* (%)				
Johns Hopkins	299 (21.7)	297 (21.9)	2 (10.0)	151 (30.4)
AI DuPont	384 (28.0)	381 (28.1)	3 (15.0)	161 (32.5)
Texas	218 (15.9)	210 (15.5)	8 (40.0)	27 (5.4)
Wisconsin	473 (34.4)	466 (34.4)	7 (35.0)	157 (31.7)
Inheritance, *n* (%)				
De novo	1,044 (76.0)	1,029 (76.0)	15 (75.0)	392 (79.0)
Inherited	191 (13.9)	190 (14.0)	1 (5.0)	73 (14.7)
Unknown	139 (10.1)	135 (10.0)	4 (20.0)	31 (6.3)
Adopted, *n* (%)	86 (6.3)			41 (8.3)
Gestational, *n* (%)				
Preterm	174 (12.7)	174 (12.9)	0	71 (14.3)
Term	1,008 (73.5)	997 (73.6)	11 (55.0)	379 (76.4)
Post term	15 (1.1)	15 (1.1)	0	1 (0.2)
Unknown	177 (12.8)	168 (12.4)	9 (45.0)	45 (9.1)
Limb lengthening, *n* (%)	17 (1.2)	17 (1.3)	0	5 (1.0)
GH deficiency, *n* (%)	4^a^ (0.3)	4 (0.3)	0	2 (0.4)
GH treatment, *n* (%)	2^b^ (0.1)	2 (01)	0	0
Trial participants, *n* (%)	12 (0.8)	12 (0.8)	0	12 (2.4)

In the PAC, 34.4% of the subjects are from the Wisconsin site, 28.0% from duPont, 21.7% from Johns Hopkins, and 15.9% from Texas. Compared with the other sites, subjects from Texas are older and more have been lost to follow-up. The active subcohort includes 5.4% from the Texas site and the rest distributed fairly evenly over the other three study sites. Unknown inheritance cannot be resolved as this was either not recorded or the medical records are missing.

*GH*  growth hormone, *IQR *  interquartile range.

^a^None of the four subjects with growth hormone deficiency were treated with growth hormone.

^b^Two subjects treated with growth hormone were not confirmed to be growth hormone deficient by provocative testing.

The PAC population, shown In Table 2, is distributed over the decades while 83.9% of the active subjects were born in the last two decades. There is limited information about the timing and precise clinical means (e.g., radiographs, family history, ultrasound) of diagnosis in the oldest PAC members due to missing records and/or loss to follow-up; this is reflected in a higher proportion of “unknown” mode of diagnosis. Regardless, all individuals included in the PAC had a clear diagnosis of achondroplasia as determined by a clinical geneticist. A lack of molecular diagnoses in the oldest cohorts is attributable to the pathogenic variant in *FGFR3* being established in 1994. Over subsequent decades, there is a pattern of earlier diagnosis with 59.8% of PAC subjects born in 2010 or later diagnosed prenatally or at birth.

**Table 2:** Population characteristics by 10-year birth cohort including vital status, age at last encounter, and timing and mode of achondroplasia diagnosis.

Characteristic	Birth decade^a^					
	Before 1980	1980s	1990s	2000s	After 2010	Total
Population distribution, *n*						
PAC	234	231	314	356	239	1,374
Active	29	25	66	177	199	496
Deceased	15	3	2	0	0	20
Age at last encounter, years						
Mean	34.9	17.7	14.8	9.7	3.4	15.4
Range	0.3-79.7	0.5-36.9	0-27.3	0.4-17.3	0.0-7.4	0.0-79.7
Median (IQR)	36.6 (17.8, 50.0)	17.4 (9.9, 26.1)	16.5 (9.9, 20.1)	9.7 (7.1, 12.3)	3.2 (1.9, 4.9)	11.9 (5.6, 19.7)
Timing of diagnosis, *n* (%)						
Prenatal	0 (0)	13 (5.6)	53 (16.9)	73 (20.5)	76 (31.8)	215 (15.6)
At birth	40 (17.1)	64 (27.7)	72 (22.9)	100 (28.1)	67 (28.0)	343 (25.0)
24 hours–1 month	16 (6.8)	42 (18.2)	51 (16.2)	74 (20.8)	39 (16.4)	222 (16.2)
>1 month	51 (21.8)	39 (16.9)	86 (27.4)	80 (22.5)	45 (18.8)	301 (21.9)
Unknown	127 (54.3)	73 (31.6)	52 (16.6)	29 (8.1)	12 (5.0)	293 (21.3)
Mode of diagnosis,^b^ *n* (%)						
Molecular only	1 (0.4)	2 (0.9)	10 (3.2)	17 (4.8)	19 (7.9)	49 (3.6)
Clinical^c^ ± molecular	85 (36.3)	138 (59.7)	239 (76.1)	315 (88.5)	213 (89.1)	990 (72.1)
Unknown	148 (63.3)	91 (39.4)	65 (20.7)	24 (6.7)	7 (3.0)	335 (24.3)

*IQR* interquartile range, *PAC* primary achondroplasia cohort.

^a^Birth decade: before 1980 = before 1 January 1980; 1980s = 1 January 1980–31 December 1989; 1990s =  January 1990–31 December 1999; 2000s = 1 January 2000–31 December 2009; after 2010 = including and after 1 January 2010.

^b^Subjects presented by decade of birth, but diagnosis made at any time since birth. Unknown mode of diagnosis represents missing records and/or loss to follow-up such that the precise method of clinical diagnosis cannot be confirmed.

^c^Clinical diagnosis includes physical exam, radiographs, ultrasound, family history.

Table 3 indicates the number of length/height, weight, and head circumference data points extracted from the medical records of the PAC members, as well as the number of subjects who contributed those points in each age category. Across all age categories, 1,365 (99.3%) subjects from the PAC contributed at least one anthropometric value to this database for a total of 37,016 anthropometry data points available for analysis.

**Table 3:** Number of length/height, weight, and head circumference values from subjects by age group^a^.

Anthropometry	Age at last known contact			
	<10 years	≥10 to 18 years	≥18 years	Total
	Points/subjects	Points/subjects	Points/subjects	Points/subjects
Length/height	4,725/565	4,466/369	3,553/384	12,744/1,318
Weight	5,582/555	5,160/361	4,217/376	14,959/1,292
Head circumference	3,610/558	3,269/366	2,434/315	9,313/1,239
Total	13,917/579	12,895/374	10,204/412	37,016/1,365

In the youngest age category (<10 years), there were 579 subjects (42.1%) of the primary achondroplasia cohort (PAC) who contributed a total of 13,917 anthropometric values. There were 374 (27.2%) individuals of the PAC contributing 12,895 anthropometry values from 10 years of age up to 18 years, and 10,204 data points from 412 (30.0%) individuals 18 years of age and older.

^a^Nine PAC members were excluded from this table (and growth curves). Five subjects were preterm and had anthropometry available only from the first 2 years of life and four had anthropometry available only after undergoing limb lengthening, growth hormone treatment, or clinical trial participation.

Separate curves were generated for length/height and weight for subjects 0–3 years of age and 0–18 years of age by sex. Scatterplots of the raw data from achondroplasia subjects with 5th, 50th, and 95th percentiles are shown along with 5th, 50th, and 95th percentile average stature reference curves in Supplemental Figures 1 and 2 for length/height and weight, respectively. WHO reference curves are included in the length and weight figures for 0–3 years of age.^22^ CDC 2000 reference curves are superimposed on the 0–18 year achondroplasia height and weight curves.^23^

As shown in Figure S1, birth length of achondroplasia subjects overlaps that of average stature newborns. Although visually comparable, the achondroplasia cohort’s average birth length is significantly lower than that of average stature newborns in both sexes (females: PAC 47.28 cm ± 2.85 cm [95% CI 47, 47.5] vs. WHO 49.1 cm ± 1.86 cm; males: PAC 47.90 cm ± 3.18 cm [95% CI 47.6, 48.2] vs. WHO 49.9 cm ± 1.89 cm).^22^ Shortly thereafter, the length curves rapidly diverge so that the 95th percentile line for achondroplasia falls below the 5th percentile line for average stature by 3 months of age in males and 4 months in females. Examination of height trajectory reveals height velocity decreases to 1 cm/year at 18.19 years of age in males and 15.43 years in females and reaches zero at 19.37 years and 18.44 years of age in males and females, respectively.

In comparison to average stature infants, the birth weight of term male and female infants with achondroplasia appears to overlap in Figure S2. However, by sex, the average birth weight for females and males with achondroplasia is actually greater than the WHO reference population (females: PAC 3.32 kg ± 0.4 kg [95% CI 3.29, 3.35] vs. WHO 3.2 kg ± 0.5 kg; males: PAC 3.41 kg ± 0.5 kg [95% CI 3.16, 3.24] vs. WHO 3.3 kg ± 0.5 kg).^22^ By ~3 months of age, the achondroplasia curves diverge below the reference population although there is persistent overlap through the teenage years.

Figure S3 shows the head circumference data from birth through 5 years of age for males and females including the 5th, 50th, and 95th percentiles with the average stature US population superimposed. To describe this entire cohort, regardless of surgical intervention involving the foramen magnum, upper cervical cord, or ventricular shunting, all raw data points were included. At birth, there is overlap of some achondroplasia birth head circumference values with the average stature reference population^22^ but many exceed the 95% CI. The mean birth head circumference for the male achondroplasia cohort was 37.1 cm ± 3.2 cm (95% CI 36.8, 37.3) compared with 34.5 cm ± 1.3 cm for average stature males, and the mean birth head circumference of females with achondroplasia was 36.4 cm ± 2.3 cm (95% CI 36.2, 36.6) compared with 33.9 cm ± 1.2 cm for average stature females.

The surgical burden of 1,374 subjects with achondroplasia is shown in Table 4. There were 1,094 (79.6%) in the PAC who had at least one surgical procedure in the five achondroplasia-related surgical categories of ENT, brain, foramen magnum, spine, and extremities. Of these 1,094 subjects, 594 (54.3%) had one achondroplasia-related surgery, 359 (32.8%) had two, 103 (9.4%) had three, 29 (2.7%) had four, and 9 (0.8%) had at least one surgical procedure in all five categories. Of the remaining 280 subjects in the PAC, 167 (12.2%) never had an achondroplasia-related surgery and the surgical history is unknown for the remaining 113 subjects (8.2%). To reduce bias caused by incomplete data (though likely underestimating the overall surgical burden), the total PAC population of 1,374 subjects was used as the denominator in Table 4 (vs. 1,261 with a known surgical history or 1,094 with at least one prior achondroplasia-related surgery). By surgical subtype, nearly two-thirds (65.0%) of the PAC had at least one of the 2,803 defined ENT procedures while 10.0% had at least one of the brain surgeries. Two hundred eighty-one individuals (20.5%) of the PAC had 314 total foramen magnum procedures. For the remainder of the spine (C3-sacrum), 175 individuals (12.7%) had 425 total surgical procedures and there were 291 subjects (22.0%) who underwent 684 surgical procedures on the extremities.

**Table 4:** Achondroplasia-related surgical categories in primary achondroplasia cohort (PAC) (*n* = 1,374).

≥1 Achondroplasia-related surgery	Procedures	Subjects, *n* (%)
Yes (ever)	4,552	1,094 (79.6)
No (never)		167 (12.2)
Unknown		113 (8.2)
Total		1,374
Surgical category^a^		
Ear, nose, and throat	2,803	893 (65.0)
Brain	326	137 (10.0)
Foramen magnum + C1/C2	314	281 (20.5)
Spine^b^	425	175 (12.7)
Extremity	684	291 (21.2)

One thousand ninety-four (79.6%) of the primary achondroplasia cohort (PAC) had at least one achondroplasia-related surgery in one of the categories and 167 (12.2%) subjects were confirmed to never have had a surgical procedure in one of the categories. The surgical status of the remaining 113 subjects (8.2%) is unknown. One hundred one subjects of 113 with an unknown surgical history were from the oldest three birth cohorts (before 1980, *n* = 37; 1980–1990, *n* = 30; 1990–2000, *n* = 34) for whom clinical records were limited and/or subjects were lost to follow-up. Thus, there are only 12 subjects from the last two birth cohorts for whom a validated surgical history is unknown. A total of 460 non-achondroplasia-related surgeries (e.g., appendectomy, Cesarean section, exploratory laparotomy) in 302 PAC subjects were extracted from the medical records. Further analysis of these procedures is not feasible because of incomplete ascertainment of all members of the PAC. Among the 302 individuals who had a non-achondroplasia-related surgery, 271 also had a least one achondroplasia-related surgery, leaving 31 subjects (31/1,374 = 2.3%) who had exclusively non-achondroplasia-related surgeries documented in their medical history.

^a^Subjects may have surgeries in more than one surgical category.

^b^Spine category includes procedures from C3 through the sacrum.

As shown in Table 5, there is a trend of more PSG being performed in recent birth decades and at a younger average age. There are 677 subjects in the PAC (49.3%) with 1,631 polysomnograms available for study. Moderate to severe OSA was found in 38.4% of all subjects with at least one prior PSG.

**Table 5:** Sleep disordered breathing by birth decade.

	Before 1980	1980–1990	1990–2000	2000-–2010	After 2010	Total
Subjects born in decade, *n*	234	231	314	356	239	1,374
Obstructive sleep apnea diagnosis, *n*						
Confirmed by polysomnography	26	31	72	164	138	429 (31.2)
Denied by polysomnography	10	31	44	63	46	195 (14.2)
Inconclusive polysomnography	7	32	13	8	5	38 (2.8)
Suspected by medical history	13	22	53	32	11	131 (9.5)
Unknown	178	141	132	89	41	581 (42.3)
Age first polysomnography, years, mean ± SD	31.1 ± 16.9	5.6 ± 7.9	3.0 ± 4.2	2.2 ± 3.1	0.8 ± 0.9	3.8 ± 8.4
≥1 polysomnography, *n* (%)	35 (15.0)	79 (34.2)	127 (40.4)	242 (68.0)	194 (81.2)	677 (49.3)
Subjects with moderate or severe OSA						
≥1 polysomnography, *n* (%)	16 (45.7)	16 (20.3)	38 (30.0)	99 (40.9)	91 (46.9)	260 (38.4)

*OSA* obstructive sleep apnea.

Finally, 1,251 subjects (91.0%) from the PAC had at least one of the following radiographic studies in their medical record: X-ray (*n* = 7,201), CT (*n* = 1,069), MRI (*n* = 1,826), echocardiogram (*n* = 166), and ultrasound (*n* = 465) for a total of 10,727 radiographic studies. X-rays primarily involved the long bones, spine, and cranium. CTs, MRIs, and ultrasounds were primarily of the brain and/or cervicomedullary junction (CMJ). Images from 75.5% of these studies are available for further primary examination. Studies performed shortly before and after a surgical procedure (e.g., MRI of brain and CMJ before and after cervicomedullary decompression [CMD]) are especially valuable and are under review.

## DISCUSSION

The results of this large US achondroplasia cohort study spanning over 40 years of follow-up data will improve anticipatory guidance and patient care. From a descriptive standpoint, the de novo pathologic variant rate of achondroplasia in the PAC is 76.0%, similar to prior reports of 80%.^6^ The proportion of adopted children in the PAC (6.3%) and active subcohort (9.3%) is higher than the 2.5% US general adoption rate but lower than the 12% adoption rate in a US specialty cleft clinic.^28^ A greater proportion of the PAC (12.7%; 174/1,374) was premature (gestation <37 weeks) than in the US population (9.8%),^29^ perhaps attributable to the shorter maternal achondroplasia trunk^30,31^ or fetal macrocephaly; both may cause cephalopelvic disproportion and prompt Cesarean section.^32^ Of the 174 premature subjects, 19 (10.9%) were born to a short stature mother while the majority were born to an average stature mother. Further study is needed to quantify the prevalence of premature labor and understand the decision to induce labor (e.g., to avoid dystocia) in average stature mothers of infants with achondroplasia.

By birth cohort, fewer prenatal diagnoses in the oldest subcohort are expected since prenatal ultrasound was new in the 1970s/early 1980s. Low image resolution precluded identification of skeletal anomalies suggestive of achondroplasia (e.g., frontal bossing, recessed nasal bridge, metaphyseal flaring). Prenatal genetic testing was not possible until the pathologic variant in *FGFR3* causing achondroplasia was identified in 1994.^4,5,33,34^ Technological advancement in amniocentesis and chorionic villus sampling also were needed to execute molecular studies. The evolution of molecular testing is reflected in the increasing proportion of molecular diagnosis, such that almost 60% of the youngest birth cohort was diagnosed prenatally or at birth. With the expansion of noninvasive prenatal testing, prenatal diagnoses will increase. Further study of the effect of greater prenatal diagnosis on the US and global live birth rate of infants with achondroplasia is warranted. Coi et al. recently postulated that earlier prenatal diagnosis could contribute to higher pregnancy termination rates in some European countries.^1^ Novel growth modulating pharmaceuticals may have an opposite effect on the achondroplasia live birth rate and access to (or lack of) a supportive community through organizations like the Little People of America may have its own influence. These factors should be compared across countries with variable access to medical care and different attitudes toward prenatal testing and pregnancy.

Examination of the anthropometry indicates linear growth deviates from average stature by 6 months of age though weight overlaps with age-matched average stature peers. Longer longitudinal study of these trends into adulthood is needed to determine if excessive weight correlates with negative health outcomes, such as hypertension or coronary heart disease, in achondroplasia as in average stature. Identification in childhood of those at risk of adult obesity would be extremely helpful for early intervention. Further research is needed to correlate body mass index with body composition and long-term health outcomes in achondroplasia.^19^

In the PAC, as in a recent European study,^30^ the achondroplasia head circumference slope is steeper than average stature and continues until ~1 year when it begins to level. Ninety percent of the head circumference is achieved by 11.1 and 11.2 months in males and females, respectively. This rapid head growth in year 1 highlights the need to monitor longitudinal head circumference to detect abnormal growth indicating increased intracranial pressure. Achondroplasia-specific head circumference curves for clinical use should represent a large population, but herein lies an important consideration: should these curves include all population measurements or exclude individuals who had CMD or shunt placement? To date, inclusion criteria for published OFC curves have varied.^30,35–37^ Having surgical history concurrent with head circumference data allows us to perform sensitivity analyses and examine patterns of growth in individuals with and without these surgeries. These issues are essential to consider in our future analysis to construct new head circumference curves, which will address the influence such neurocranial procedures may have on growth.

Our surgical data show evidence of a heavy surgical burden in achondroplasia. Nearly 80% had at least one surgery in one of the five achondroplasia-related categories. To compare, only tonsillectomy/adenoidectomy and pressure equalizing tubes are performed in average stature children, but at a far lower rate. For example, 56.1% of the PAC had one or more sets of PE tubes compared with only 6.8% of the average stature population by 3 years.^38^ Considering adenotonsillectomy, 53.5% of the PAC had this procedure compared with 0.7–2% of the general population by 15 years.^39^

In this US achondroplasia cohort, surgical limb-lengthening prevalence is 1.2%. For comparison, the impression of the director of a Japanese academic surgical clinic is that ~60% of his achondroplasia population undergoes lower limb lengthening (personal communication, Dr. Natsuo Yasui). In a Spanish academic surgical clinic, lower limb-lengthening prevalence is estimated at ~90% with an increasing interest in upper limb lengthening (personal communication Dr. Antonio Leiva Gea). These data should be interpreted with the caveats that they are derived from surgical clinics (vs. genetic medicine) in large academic centers and there is no national clinical database to ascertain broader population prevalence data. Unlike Japan, where growth hormone has been an approved treatment for achondroplasia since 1997, there were only two subjects in the PAC who had received this treatment. Multicenter clinical outcome studies to examine physical function, pain, and psychological health that result from these varied treatments, as well as cultural attitudes and beliefs about these procedures, are needed.

The last two study domains addressed are sleep disordered breathing, as documented by PSG, and a radiographic catalogue of all available studies. Regarding the former, 38.4% of the PAC have moderate to severe OSA. In average stature, the prevalence estimate is 5% in children,^15^ and 22% and 17% in adult men and women, respectively.^40^ There are more polysomnograms available in recent decades as awareness of sleep disordered breathing in achondroplasia increased. In the final study domain, we have over 10,700 radiographic studies available to correlate with surgical outcomes (e.g., results of C-spine MRI and PSG before and after CMD). Clinically active patients can be further examined for their physical function and contrasted with subjects who did not undergo these surgical procedures.

The main strengths of this study are the size of this natural history cohort, the follow-up of over four decades, and the uniform data collection by medical providers with extensive achondroplasia care experience. This well-phenotyped cohort may serve as a natural history control group to those undergoing novel drug or surgical interventions. This study also demonstrates feasibility of clinical data collection across multiple institutions in a uniform manner. We continue to explore these data with multivariate analysis and plan to expand this study to compare global differences in health-care resources and practices. The knowledge gained will be invaluable to patients, their families, and healthcare providers everywhere to better prepare for complications and improve clinical outcomes.

## Supplementary information

Supplemental Methods

## Data Availability

Data presented in this paper are available upon request of the authors.

## Code availability

The statistical code written for this analysis pertains only to this database and is accessible to study team members from the secured servers housing the database.
