# Supplementary material for: Achondroplasia Natural History Study (CLARITY): a multicenter retrospective cohort study of achondroplasia in the United States
Source: Genet Med. 2021 May 18;23(8):1498–505. doi: 10.1038/s41436-021-01165-2 (PMC8354851; doi:10.1038/s41436-021-01165-2)
Supplement: Supplementary file 1 — Supplemental Methods [file 41436_2021_1165_MOESM1_ESM.docx]

**Supplemental Methods**

**Participating sites and study population**

Subjects were evaluated at least once between 1957 and 2017 at one or more of four academic skeletal dysplasia centers: Johns Hopkins University Greenberg Center for Skeletal Dysplasias (Baltimore, MD)-coordinating site, AI duPont Hospital for Children (AIDHC) (Wilmington, DE), McGovern Medical School at the University of Texas Health (Houston, TX), and University of Wisconsin (Madison, WI). All four academic sites have a history of active engagement and long-standing commitment to large achondroplasia patient populations. The diagnosis of achondroplasia was made by molecular or clinical means (e.g., physical exam, radiographs, family history) by one of the study co-PIs or a local clinical geneticist. If there was ambiguity regarding the diagnosis (e.g. possible hypochondroplasia and no molecular test result available to verify the diagnosis), that subject was excluded. Also excluded were patients with compound heterozygosity, homozygosity, or double heterozygosity for two bone growth disorders.

All subjects evaluated at the four clinical sites for medical care and for whom medical records were available are collectively considered the primary achondroplasia cohort (PAC) for the present analyses. Patients who sought clinical care at any of the four sites over the period of data collection signed a locally-approved informed consent form with site study personnel; these individuals are identified as the active sub-cohort and will be approached for participation in future studies under a separate IRB-approved protocol. Data from subjects who were lost to follow-up at any of the clinical sites were abstracted under a waiver of consent. This process was approved by the local Institutional Review Board at each participating institution.

**Data collection and management**

All available retrospective data were extracted from archives and current hard-copy and electronic medical records at each study site to populate a REDCap database (i.e. web-based application designed for clinical research data entry). All primary data are maintained at the clinical site from which it originated. Only the REDCap administrator and the data manager/biostatistician at the coordinating site have electronic access to the complete database. Otherwise, study personnel from each site have access only to their data. This REDCap database is hosted on servers administered by the Data Informatics Services Core (DISC) of the Johns Hopkins Biostatistics Center. Multi-user data entry can occur simultaneously, and data are backed up regularly with storage off-site. The database was designed to add new fields for new prospective studies and to easily add more sites.

Uniform data collection forms were created to ensure similar data collection at all study sites. Definitions of data fields and a question-by-question (QXQ) document were compiled in a Manual of Operations and distributed to all sites. Modifications to data collection fields were discussed in bi-weekly conference calls and three-times per year in-person meetings. Queries of potential data entry errors or omissions were communicated from the coordinating site to the coordinator at each study site. Corrections were resolved by the primary data entry site until the database was closed to new entries in January 2019 after which changes were only made by the coordinating site as needed to resolve remaining issues. The database was closed in May 2019 for the analysis presented here.

Data were monitored and cleaned throughout the entry process for potential inclusion of a subject at more than one site. When detected, the coordinating site examined the raw data for duplication of data values. Unique data entered by more than one study site for the same subject were merged for analysis. Identical data values entered by more than one study site for the same subject were deleted from the site with the least total data entered for that subject. Thus, only one copy of duplicated data values remained for analysis.

Unless otherwise noted, the age of a subject reflects his/her age at the last known clinical encounter (in person or via medical records). All anthropometry was collected by multiple providers in the clinic setting and recorded in the hard-copy or electronic medical record. Length was obtained in a supine position until at least 2 years of age when the majority of individuals with achondroplasia can and could participate in a standing height measurement with a stadiometer. Weight was obtained with a standard clinical scale and head circumference was measured by a physician or nurse in the dysplasia center with a tape measure. When populating the REDCap database, anthropometry values were extracted from all available clinical records along with the date the measurement was made. Once compiled, values were visually examined for outliers and implausibilities by plotting each parameter (length/height, weight, and head circumference) over time on individual and cohort liner plots. The following algorithms also were applied to the data cleaning process of all anthropometry values to identify potential erroneous data values: length/height difference greater than 10 cm between 2 values obtained within 2 months, length/height difference greater than 5 cm between any 2 data points, height over 139.9 cm, weight over 69 kg, and weight difference greater than 5 kg between 2 values obtained within 6 months. All head circumference data from 0 to 5 years of age were examined for values over 60 cm and a change of more than 3 cm within 6 months. Additionally, length/height, weight, or head circumference values obtained on 2 or more visits occurring less than 15 days apart were averaged and a single value was assigned to represent the values. Discrepancies were resolved by checking the value in question against the primary data source. If the primary data source was found to include a value that was physiologically implausible, and therefore reflecting a prior error in measurement or recording, that value was deleted from analysis.

Operative notes, admission/discharge notes, and clinical notes from available medical records were examined for documentation of all surgical procedures and the date performed. When recorded, the indication for the surgical procedure, hospital location where the procedure took place, and outcomes of the procedure were noted. For analysis, there were several surgical procedures considered together under each of the 5 main categories: ear-nose-throat (ENT), brain, foramen magnum, spine, and extremities. The primary tonsillectomy/adenoidectomy and all revisions were included in the ENT category. Surgical placement of pressure equalizing (PE) tubes was included in the analysis only if a date of the procedure was available. This was to avoid “double counting” PE tube placement as an independent surgical event when it actually occurred concurrently with adenotonsillectomy. Therefore, this likely underestimates the total number of PE tubes placed surgically in this cohort but was preferable to overestimating the events. In the brain surgery category, all occurrences of ventriculoperitoneal shunt placement and revision as well as ventriculostomy are included. The third surgical category involves enlargement of the foramen magnum with/without laminectomy of the first and sometimes second cervical vertebrae. These procedures are considered together in the foramen magnum category but separate from laminectomies and vertebral fusions of cervical vertebra 3 (C3) through the sacrum in the fourth category of spine surgery. The extremity surgery category includes a variety of procedures to correct genu varum deformity and lower extremity bowing that is relatively common and characteristic of achondroplasia. Other medical conditions (e.g., craniosynostosis) were compiled from available medical records and based on the presence of the condition at the time of the last known medical encounter.

All available polysomnography (PSG) reports were identified in the hard-copy and electronic medical record for each subject. When available, the following parameters from the PSG reports were entered into the database: indication for the study, date performed, number of channels used for the study, time in bed, total sleep time, use of room air or supplemental oxygen, saturation nadir, maximum end-tidal CO2 and percentage of time elevated, number of obstructive, hypopneic, and central events, length of central apneas and presence of accompanying desaturation, absolute and percentage of time spent in rapid eye movement sleep, number of arousals and the diagnostic determination of the presence (or lack), and severity of obstructive and central sleep apnea. The final determination of individuals with at least one sleep study with moderate or severe obstructive sleep apnea (OSA) was included in this analysis. The results of the first query in the medical history of each subject was also included: “has the participant ever been diagnosed with OSA?” This was further specified as confirmed, denied, or inconclusive by PSG; suspected based on medical history, or unknown. This response was a clinical determination from the PI and coordinator at each site at the time of data entry.

The final study domain was construction of a radiographic catalogue of all x-rays, computed tomography scans, magnetic resonance imaging scans, echocardiograms, and ultrasounds performed on all subjects from the medical records. For each encounter, the type of study was recorded, the date performed, body part included in the imaging, the medical facility of the imaging, and whether the original images were available for evaluation from a hard copy of films, a CD, or electronically. If the images were no longer available for review, this was also recorded as reports of the imaging are not considered sufficient for analysis.

**Supplemental Analysis**

Specific to the anthropometry analysis, the number of length, weight, and head circumference data points are shown for PAC subjects by age group in tabular form (Table 3 in manuscript); subjects known to be born before 37 weeks gestation (i.e., preterm) were omitted from the corresponding growth curves up to 2 years of age due to the effect of prematurity on size and growth rates. This is in accordance with the practice in average stature WHO and CDC reference populations. All anthropometric data points from subjects born post-term are included in all growth curves. The birth parameters (i.e. length, weight, and head circumference) of subjects with unknown gestational age were examined for their percentile position on published sex-specific achondroplasia growth curves. If within ±2 SD of the mean at birth, that individual was presumed to have been born at term and all their anthropometry were included in the analysis from birth onwards.

Additional considerations for the anthropometry data pertain to subjects who underwent limb lengthening, were treated with growth hormone (with or without documented deficiency), or participated in a clinical trial involving an investigational pharmaceutical designed to modulate linear growth. For individuals in any of these three groups, growth data (including length/height, weight, and head circumference) *after* limb lengthening, *after* implementation of growth hormone treatment, and/or *after* enrollment in a clinical trial were excluded from analysis in this and subsequent manuscripts; all other clinical data were included.

Similar to that utilized in our prior publication of a smaller achondroplasia cohort, sex-specific percentile curves were generated in a 2-step process for children with anthropometry data up to 18 years of age for stature and weight and from birth through 5 years for head circumference. First, empirical 5^th^, 50^th^, and 95^th^ percentiles were computed using values within an age-specific window-based approach at each month of age for the period of 0-18 years. For the first year of age (0 to 12 months), a window of ±0.5 months was used to estimate the empirical month-specific percentile; for 1 to 3 years (greater than 12 to 36 months), a window of ±1 month was used; for 3 to 10 years (greater than 36 months to 120 months), a window of ±3 months was used; and for 10 to 18 years (121 to 216 months), a window of ±6 months was used. While the percentile curve estimates for the anthropometry measures were robust to the choice of smoothing windows, the data were more densely concentrated in younger subjects and the narrower windows in these younger age groups allowed for more nuanced estimates of the empirical percentiles.

The height and OFC data were relatively symmetrically distributed across the analyses across 0 to18 years and 0 to 5 years of age, respectively. As such, all data between 0 and 18 years for length/height and 0 to 5 years for occipitofrontal circumference (OFC) were used to estimate penalized smoothing splines to create isopleths for stature for age and head circumference for age for the 5th, 50^th^, and 95^th^ percentiles. Smoothing parameters, including the optimal number of knots, were chosen by cross-validation. Because of the relative skewness of weight for age data, with skewness increasing with increasing age, separate penalized smoothing splines were used to estimate the weight for age percentile curves for the ages from 0 to 3 years and 3 to 18 years. The number of knots were adjusted to balance model fit with enforced monotonicity of the weight for age percentile estimates.

For the height-velocity by age analysis, only the mean height-velocity by age was estimated for the period from age 0 to 25 years. The same exclusion of age 0-2 years measurements for children born prematurely was used for the height velocity analyses. For all subjects, the height-velocity at a given age was computed by taking the difference in two time-ordered length/height values and dividing this by the difference in ages of measurements, and only estimates based on length/height values measured between 6 and 18 months apart were used to construct the sex-specific height velocity by age curves.

Sex-specific birth mean and standard deviation (SD) estimates and 95% confidence intervals (CIs) were computed for length, weight, and OFC. These resulting CIs were compared to the same average-stature mean values from CDC/WHO to ascertain whether these differences were statistically significant.

Table S1. Summary of 20 deceased subjects in primary achondroplasia cohort (PAC)

| Sex | Birth cohort | Age at death  (years) | Cause of Death | Description |
| --- | --- | --- | --- | --- |
| F | 1980a | 26.7 | Unknown |  |
| F | <1980s | 30.9 | Unknown |  |
| M | <1980s | 58.6 | Unknown |  |
| F | <1980s | 74.8 | Unknown |  |
| F | <1980s | 64.9 | Unknown |  |
| M | <1980 | 77.8 | Unknown |  |
| F | <1980s | 13.6 | Post-operative complications | Unknown surgery type; cause of death was pulmonary edema, acidosis, coagulopathy |
| F | <1980s | 44.3 | Post-operative complications | Unknown surgery type and specific complications |
| M | <1980s | 50.1 | Post-operative complication | Surgery for bleeding gastric ulcer; cause of death was hemorrhage |
| F | <1980s | 68.9 | Post-operative stroke | Surgery was T1-T2 laminectomy/fusion for kyphosis; cause of death was pontine and right thalamic stroke |
| F | 1980s | 2.7 | Pneumonia | NOS |
| F | <1980s | 16.9 | Aspiration pneumonia | NOS |
| M | <1980s | 50.0 | Pneumonia | NOS |
| M | 1990s | 2.6 | Apnea | Presumed central apnea due to cervicomedullary compression |
| F | <1980s | 38.8 | Apnea | Due to dysrhythmia |
| M | <1980s | 0.3 | SIDS | Before surveillance recommendation with MRI/CT/sleep study |
| M | 1990s | 19.3 | Multiple trauma | Motor vehicle accident |
| F | 1980s | 22.7 | Cerebral hemorrhage | Attributed to chronic meningitis |
| F | <1980s | 45.1 | Seizure | Drowned in bathtub |
| M | <1980s | 79.7 | Natural causes |  |

Of the 14 deceased individuals with clinical information, 4 died from post-operative complications, 3 others from pneumonia, and 2 attributed to apnea (a dysrhythmia and central apnea due to cervical cord compression). The cause of death of the remaining 5 individuals was recorded as noted. NOS = not otherwise specified; SIDS = sudden infant death syndrome.

**Figure S1. Achondroplasia length-for-age 0-36 months (top graphs), and height-for-age 0-18 years (bottom graphs)**.

Length-for-age curve for achondroplasia males (left top) includes 2,971 observations from 541 subjects. Achondroplasia females (right top) includes 2,682 observations from 488 subjects. WHO reference curves for age-matched, sex-matched children are superimposed.

Height-for-age curve for males (left bottom) includes 3,957 observations from 546 subjects. Achondroplasia females (right bottom) includes 3,705 observations from 502 subjects.

In Figure S1, birth length of achondroplasia subjects has large overlap with that of average stature infants. Although visually comparable, the achondroplasia cohort average birth length is significantly lower than that of average stature newborns in both sexes (females: PAC 47.28 cm ± 2.85 cm [95% CI 47, 47.5] versus WHO 49.1 cm ± 1.86 cm; males: PAC 47.90 cm ± 3.18 cm [95% CI 47.6, 48.2] versus WHO 49.9 cm ± 1.89 cm). Shortly thereafter, the length curves rapidly diverge so that the 95^th^ percentile line for achondroplasia falls below the 5^th^ percentile line for average stature by 3 months of age in males and 4 months in females. The upward slope of linear growth in achondroplasia is less steep, falling below average stature, sex- and age-matched subjects by early childhood and continuing throughout the teens. From analysis of height trajectory in the PAC, height velocity decreases to 1 cm/year at age 18.19 years in males and age 15.43 years in females and reaches zero at age 19.37 years and 18.44 years, in males and females, respectively.

**Figure S2**. **Achondroplasia weight-for-age 0-36 months (top graphs) and weight-for-age 0-18 years (bottom graphs).**

Weight-for-age curves for males for 0-36 months (left top) and 0-18 years (left bottom) include 3,604 observations from 550 subjects and 4,591 observations from 532 subjects, respectively. Curves for females for 0-36 months (right top) include 3,112 observations in 493 subjects and for 0-18 years (right bottom) include 4,293 observations from 486 subjects.

In comparison to average stature infants, birth weight of term male and female infants with achondroplasia appears to overlap. However, by sex, the average birth weight for females and males with achondroplasia is greater than the WHO reference population (females: PAC 3.32 kg ± 0.4 kg [95% CI 3.29, 3.35] versus WHO 3.2 kg ± 0.5 kg; males: PAC 3.41 kg ± 0.5 kg [95% CI 3.16, 3.24] versus WHO 3.3 kg ± 0.5 kg). By ~3 months of age, the achondroplasia curves diverge from the reference population although there is persistent overlap of the upper 50% of the achondroplasia weight curve with the lower 50% of the average stature weight curve through 36 months even though the length has no overlap after ~ 6 months of age. As shown in the lower half of Figure S2, the raw achondroplasia data points and 5^th^, 50^th^, and 95^th^ percentiles overlap with average stature through the teenage years.

**Figure S3**. **Head circumference-for-males age 0-5 years (top) and females (bottom) with achondroplasia compared to age-matched average stature children.**

Figure S3 shows the head circumference data from birth through age 5 years for males (535 subjects, 3,452 observations) and females (489 subjects, 3,015 observations) including the 5^th^, 50^th^, and 95^th^ percentiles with the average stature US population superimposed. All raw data points were included in the head circumference curves regardless of surgical history involving the foramen magnum, upper cervical spine, or ventricular shunting, with measurements from those born prematurely omitted. At birth, there is overlap of achondroplasia head circumference values with those of the average stature reference. However, many circumference values exceed the 95% CI for average stature at birth. This is reflected in the significantly greater head circumference in infants with achondroplasia as compared to the reference WHO population. For males, the birth head circumference for the achondroplasia cohort was 37.1 cm ± 3.2 cm (95% CI 36.8, 37.3) compared to the average stature birth head circumference of 34.5 cm ± 1.3 cm, and that of females with achondroplasia was 36.4 cm ± 2.3 cm (95% CI 36.2, 36.6) compared to average stature females of 33.9 cm ± 1.2 cm. CI = confidence interval.
